# Comparative Analysis of the Efficacy of Transcutaneous Electrical Nerve Stimulation in Somatic and Idiopathic Tinnitus Patients

**DOI:** 10.1002/brb3.70429

**Published:** 2025-03-18

**Authors:** Emre Soylemez, Aydin Sinan Apaydin, Zehra Aydoğan, Neslihan Hazal Şen, Murat Yasar, Ömer Gökay Argadal

**Affiliations:** ^1^ Department of Audiometry Karabuk University Karabuk Turkiye; ^2^ Department of Neurosurgery Karabuk University Karabuk Turkiye; ^3^ Department of Audiology Ankara University Ankara Turkiye; ^4^ Department of Physical Therapy and Rehabilitation Karabuk University Karabuk Turkiye; ^5^ Department of Otorhinolaryngology Kastamonu University Kastamonu Turkiye; ^6^ Department of Neurosurgery Kastamonu University Kastamonu Turkiye

**Keywords:** idiopathic, neck, somatic, TENS, tinnitus

## Abstract

**Objective:**

This study aimed to investigate the effectiveness of transcutaneous electrical nerve stimulation (TENS) treatment in patients with chronic somatic tinnitus (CST) originating from the neck and idiopathic chronic subjective tinnitus (ICST).

**Methods:**

The study was conducted on 21 CST and 25 ICST individuals. These individuals were divided into two groups. Active TENS therapy was applied to one group, and a placebo was applied to the other group. Tinnitus Handicap Inventory (THI), Visual Analog Scale (VAS), Beck Anxiety Inventory (BAI), and Short Form‐36 (SF‐36) were applied to the individuals before and after the therapy.

**Results:**

In the CST group that received active treatment, significant improvements were noted in THI, VAS‐tinnitus, VAS‐neck pain, and SF‐36 (energy and pain) after treatment (*p* < 0.05). In the ICST group that received active treatment, significant improvements were observed in tinnitus loudness, THI, tinnitus loudness, BAI, VAS‐Tinnitus, and SF‐36 (physical function, mental health, and physical role limitations) after treatment (*p* < 0.05).

**Conclusion:**

Although cervical TENS therapy is considered to be a more effective treatment method for neck‐related CST patients, our placebo‐controlled comparative study demonstrates that cervical TENS can be effectively used to alleviate tinnitus and improve the quality of life in both CST and ICST patients.

## Graphical abstract

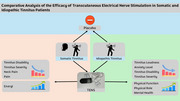

## 1 Introduction

Tinnitus, with a global prevalence ranging from 10% to 24%, is defined as the perception of sound in the ear or head without any external acoustic stimulation (Jarach et al. 2022). Ten percent of cases are reported as chronic tinnitus, while 95% are classified as subjective tinnitus (De Ridder et al. 2021). Although subjective chronic tinnitus is often associated with auditory disorders such as presbycusis, Meniere's disease, ototoxicity, and noise‐induced hearing loss, it can also occur alongside psychological disorders and somatic disorders in the head and neck region (Bauer 2018; Baguley et al. 2013; Levine 1999). However, in approximately 25% of cases, no cause can be identified, and this condition is considered idiopathic tinnitus (Byun et al. 2020). The extent to which tinnitus affects an individual's life is related to the frequency of symptom perception, severity, level of focus on it, and other accompanying health issues. Psychological disorders caused by tinnitus can reduce individuals' quality of life and increase their susceptibility to the development of chronic diseases (Tutar et al. 2020).

Despite advances in medicine and research, the pathophysiology of tinnitus is still not clearly understood. According to traditional views, sensory loss caused by otological disorders leads to an increase in central neural gain. This condition is characterized by altered bandwidth settings and increased spontaneous and synchronized neural activity (Roberts and Salvi 2019). However, many authors have recently suggested that tinnitus arises from the failure of the central auditory system to compensate for reduced sensory input (Hofmeier et al. 2018; Möhrle et al. 2019; Berlot et al. 2020). The lack of understanding of tinnitus mechanisms has resulted in inadequate and symptomatic treatment strategies.

Transcutaneous electrical nerve stimulation (TENS), an electrotherapy method, is a noninvasive technique that stimulates muscles and nerves through surface electrodes. TENS facilitates reflex activation of sympathetic inhibitory neurons and reflex inhibition of parasympathetic excitatory neurons, making it commonly used in pain management (Aydoğan et al. 2022). TENS applied to the cervical (C2) region is also frequently utilized in the treatment of tinnitus. The dorsal cochlear nucleus (DCN) receives inputs not only from auditory inputs but also directly and indirectly from the dorsal column, spinal trigeminal nuclei, dorsal raphe, and locus coeruleus (Vanneste et al. 2010; Zhang and Guan 2007; Malfatti et al. 2022). Electrical stimulation is assumed to block abnormal sensory signals by modulating the DCN (Luo et al. 2012) and improving tinnitus symptoms by increasing cochlear blood flow (Byun et al. 2020).

There are apparent similarities between tinnitus and chronic pain. Both symptoms are associated with psychological disturbances, central involvement, and hypersensitivity (De Meulemeester et al. 2023). In addition, both are subjective, can change over time, can be masked, and similar methods such as TENS can be used for treatment (Paolucci et al. 2021). Chronic tinnitus is often accompanied by temporomandibular joint pain, headache, and neck pain (Deklerck et al. 2020). In addition, these disorders can modulate tinnitus (somatic tinnitus).

Although TENS is a promising method in tinnitus treatment, success cannot be guaranteed due to the unknown pathogenesis of tinnitus. A meta‐analysis study reported that TENS therapy provided subjective symptom relief in 40% of patients (Byun et al. 2020). However, there is insufficient evidence to make TENS recommendations for specific tinnitus cases. We found only one study in the literature investigating the effectiveness of TENS according to tinnitus etiology (Steenerson and Cronin 1999). According to our hypothesis, patients with chronic somatic tinnitus (CST) associated with neck pain may benefit more from C2 TENS therapy than patients with idiopathic chronic subjective tinnitus (ICST). Therefore, this study aimed to investigate the effectiveness of TENS in patients with CST and ICST.

## 2 Materials and Methods

### 2.1 Participants and Ethical Approval

The sample size was calculated by a two dependent mean (matched pairs) test using G*Power 3.1 software. With an effect size of 1.38, a power of 80%, and a significance level of 0.05, the minimum required sample size was determined to be five participants for each intervention group (Aydoğan et al. 2022).

This study was approved by the Kastamonu University Clinical Research Ethics Committee (2024‐KAEK‐39). Written and verbal consent was obtained from all participants included in the study.

The study was conducted on patients presenting to the otorhinolaryngology clinic with complaints of subjective chronic tinnitus (> 6 months). Patients' medical histories and tinnitus information were obtained. All patients underwent a comprehensive audiological and otoneurological examination. Brain and posterior fossa magnetic resonance imaging (MRI) studies were performed on all patients to exclude objective tinnitus. Individuals with CST associated with neck pain were referred to a specialized neurosurgeon for evaluation. Patients with otological, systemic, psychiatric, and neurodegenerative diseases and those with hearing loss (pure tone hearing thresholds > 20 dB at 0.5, 1, 2, and 4 kHz) were excluded from the study. As a result, 30 participants with CST and 30 with ICST were initially included in the study. Using simple randomization, active TENS therapy was administered to 20 individuals in both groups, while 10 received a placebo (30 min daily for 4 weeks). Participants who discontinued therapy were excluded from the study

### 2.2 TENS Therapy

A Frelly (Shenzhen Kentro Medical Electronics Co. Ltd.) brand dual‐channel device was used for TENS. The electrodes in the first channel were carefully placed at the C2–C3 level in the posterior C2 region surrounding the spinal cord, and the electrodes in the second channel were carefully placed at the C5–C6 level in the same region. TENS parameters were adjusted as specified by Aydoğan et al. (2022). All participants received TENS for 30 min per day (20 sessions in total) for 4 weeks.

### 2.3 Procedure

The entire procedure applied to the participants was repeated before and after TENS therapy. The neck pain severity of the individuals in the CST group was assessed with a visual analog scale (VAS‐Neck Pain). A 10 cm line was drawn on a blank sheet of paper, and the line's starting point was numbered 0 (*no neck pain*), and the ending point was numbered 10 (*extremely severe neck pain*). The tinnitus severity of all individuals was assessed with a visual analog scale (VAS‐tinnitus). A 10 cm line was drawn on a blank sheet of paper, and the line's starting point was numbered 0 (*no tinnitus*), and the ending point was numbered 10 (*extremely severe tinnitus*). For VAS‐Neck Pain and VAS‐Tinnitus, individuals were asked to mark the severity that would be appropriate for their condition.

The neuro‐audio (Neurosoft Camp., Russia) computerized audiometry device was used for hearing assessment. The participants' air conduction hearing thresholds between 250 and 14000 Hz were determined. Bone conduction thresholds between 500 and 4000 Hz were determined. In addition, tinnitus loudness and pitch matching were performed from psychoacoustic measurements. In individuals with bilateral tinnitus, the side with more severe tinnitus was matched.

The Turkish version of the Beck Anxiety Inventory was used to assess anxiety levels (Ulusoy et al. 1998). The inventory was given to the patients, and they were asked to carefully read the 21 questions in the inventory and score the questions between 0 and 3.

The Turkish version of the Tinnitus Handicap Inventory was used to assess tinnitus handicaps (Aksoy et al. 2007). The inventory was given to patients, and they were asked to carefully read the 25 questions and score the questions as 0 (*no*), 2 (*sometimes*), or 4 (*yes*).

The Turkish version of the Short Form‐36 was used to assess quality of life (Koçyiğit et al. 1999). The inventory, consisting of eight subdimensions (physical function, physical role restriction, emotional role, energy/vitality, mental health, social functionality, pain, and general health) and 36 questions, was given to the participants. The participants were asked to read the questions carefully and answer them.

### 2.4 Statistical Analysis

IBM SPSS 21 software was used for statistical analysis. Normality distribution was checked with the Shapiro–Wilk test. Normally distributed data were presented with mean ± ss, and non‐normally distributed data were presented with median (min–max). One‐way ANOVA (more than two groups) or student *t*‐test was used to compare groups when the assumption of normality was met, and the Kruskal–Wallis test ANOVA (more than two groups) or Mann–Whitney *U* test was used when it was not met. A paired *t*‐test was used to evaluate treatment efficacy when the assumption of normality was met, and the Wilcoxon test was used when it was not met. Categorical data were compared with the chi‐square test. *p* < 0.05 was accepted as a statistical significance level in all statistical analyses.

## 3 Results

### 3.1 Initial Assessment

There was no difference between the CST and ICST groups in terms of age, gender, THI, general and psychoacoustic properties of tinnitus, and VAS‐Tinnitus (*p* > 0.05, Table 1). However, the anxiety level of individuals with CST was worse than those with ICST (*p* < 0.001, Table 2). When SF‐36 was examined, the energy and general health subscales of individuals with CST were worse than those with ICST (*p* < 0.05, Table 2). However, there was no difference in terms of other subscales (*p* > 0.05).

**TABLE 1:** Age, gender, Tinnitus Handicap Inventory (THI), and psychoacoustic findings according to idiopathic chronic subjective tinnitus (ICST) and chronic somatic tinnitus (CST) groups.

Initial assessment	CST, *n*: 21, mean ± SD or median (min–max)	ICST, *n*: 25, mean ± SD or median (min–max)	*p*
Age,	44.42 ± 12.20 (18–61)	43.48 ± 12.21 (23–63)	0.794^a^
Gender, *n*			0.845^b^
Female	12 (57.1%)	15 (60.0%)	
Male	9 (42.9%)	10 (40.0%)	0.212^c^
THI	48 (4–64)	39.28 ± 21.36	
VAS‐Tinnitus	6.00 ± 2.14	5.24 ± 1.64	0.180^a^
Tinnitus pitch (kHz, severe ear)	6 (0.25–10)	6 (0.5–8)	0.777^c^
Tinnitus loudness (dB, severe ear)	40 (30–75)	40.80 ± 16.75	0.850^c^
Tinnitus type			0.635^b^
High‐pitched ringing	12 (57.1%)	16 (64.0%)	
Noise	9 (42.9%)	9 (36.0%)	
Tinnitus Duration, years	2 (1–7)	3.76 ± 2.04	0.263^c^
Tinnitus Lateralization			0.403^b^
Left	3 (14.3%)	7 (28.0%)	
Right	6 (28.6%)	4 (16.0%)	
Bilateral	12 (57.1%)	14 (56.0%)	

Abbreviation: VAS: visual analogue scale.

^a^ Student *t* test.

^b^ Chi‐square Test.

^c^ Mann Whitney *U* test.

**TABLE 2:** Back Anxiety Inventory (BAI) and Short Form‐36 (SF‐36) findings according to idiopathic chronic subjective tinnitus (ICST) and chronic somatic tinnitus (CST) groups.

Initial assessment	CST, *n*: 21, mean ± SD or median (min–max)	ICST, *n*: 25, mean ± SD or median (min‐max)	*p*
**BAI**	19.14 ± 8.29	10.36 ± 3.53	< 0.001^a^
**SF‐36**			
Physical function	70 (35–100)	70 (35–100)	0.409^b^
Physical role restriction	25 (0–100)	50 (25–100)	0.107^b^
Emotional role	0 (0–100)	66 (0–100)	0.101^b^
Energy/vitality	32.85 ± 18.67	65 (25–80)	< 0.001^b^
Mental health	50.33 ± 19.20	64 (48–88)	0.017^b^
Social functionality	59.64 ± 52.12	64.00 ± 15.44	0.475^a^
Pain	45.00 ± 26.82	53.20 ± 14.81	0.197^a^
General health	47.61 ± 14.54	64.80 ± 15.30	< 0.001^a^

^a^ Student *t*‐test.

^b^ Mann‐Whitney *U* test.

### 3.2 Effect of TENS Therapy

Of the patients with ICST, 25 (83.33%) completed the treatment. Of these, 18 (72%) were in the active TENS therapy group (ICST‐T), and 7 (28%) were in the placebo group (ICST‐P). Of the patients with CST, 21 (70%) completed the treatment. Of these, 15 (71.4%) were in the TENS therapy group (CST‐T), and 6 (28.6%) were in the placebo group (CST‐P). There was no difference between the groups in terms of age and gender (*p* > 0.05). The distribution of age and gender by groups is presented in Table 3.

**TABLE 3:** Age and gender distribution in groups.

	CST‐T, *n*: 15	CST‐P, *n*: 6	ICST‐T, *n*: 18	ICST‐P, *n*: 7	*p*
Age, mean ± SD. (min–max)	44.93 ± 12.41 (19–61)	43.16 ± 12.71 (18–53)	42.55 ± 11.98 (23–63)	45.85 ± 13.45 (24–61)	0.920^a^
Gender, *n*					0.707^b^
Female	9 (60.0%)	3 (50%)	12 (66.6%)	3 (42.9%)	
Male	6 (40.0%)	3 (50%)	6 (33.3%)	4 (57.1%)	

Abbreviations: CST‐P: Chronic somatic tinnitus‐Placebo; CST‐T, chronic somatic tinnitus‐TENS; ICST‐P, idiopathic chronic subjective tinnitus‐placebo; ICST‐T, idiopathic chronic subjective tinnitus‐TENS.

^a^ Student *t* test.

^b^ Chi‐square Test.

According to the C2 MRI results, all patients with CST had neck problems that did not require surgery, attributed to either the flattening of C2 lordosis or the onset of C2 disc herniation. The neck pain severity (VAS‐neck pain) of individuals in the CST‐T group was 7.73 ± 1.57, and the mean neck pain duration was 8.86 ± 2.92. The neck pain severity of individuals in the CST‐P group was 6.00 ± 2.60, and the mean neck pain duration was 3.83 ± 1.16. There was no difference in neck pain severity and duration between the groups (*p* = 0.075, 0.733, respectively).

**FIGURE 1:**
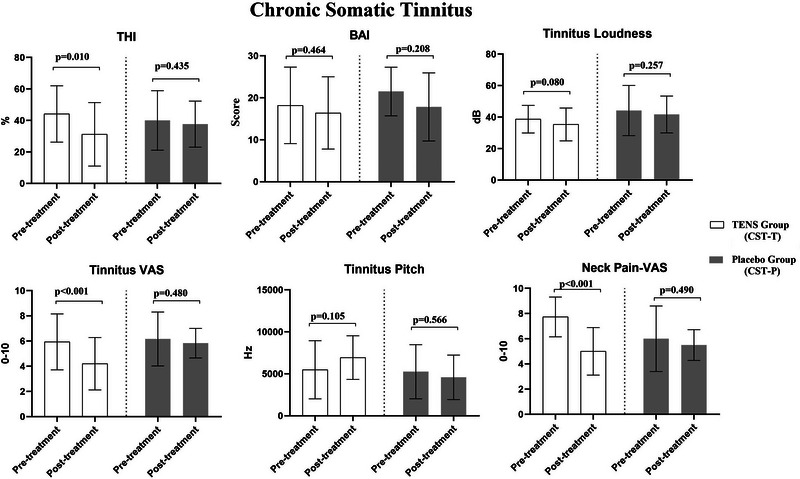
The effects of TENS (CST‐T) and placebo (CST‐P) on tinnitus handicap level, anxiety level, tinnitus‐VAS, neck pain‐VAS, and psychoacoustic characteristics in the chronic somatic tinnitus group.

**FIGURE 2:**
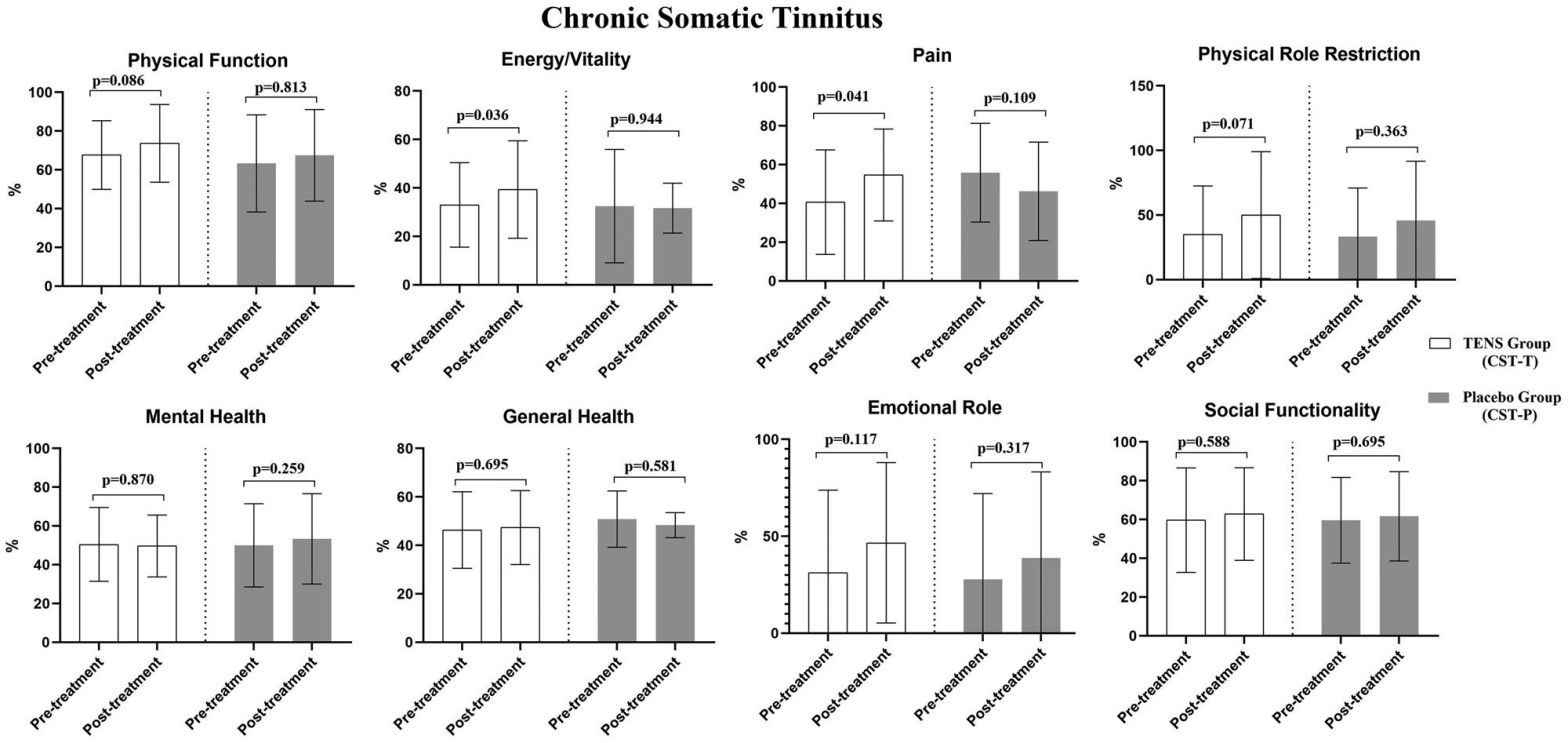
The effects of TENS (CST‐T) and placebo (CST‐P) on SF‐36 in the chronic somatic tinnitus group.

In the CST‐T group, significant improvements were observed in THI, VAS‐Tinnitus, VAS‐Neck Pain, and SF‐36/Pain scores following TENS therapy (*p* < 0.05). However, no changes were found in other parameters before and after the therapy (*p* > 0.05, Figures 1 and 2). In the CST‐P group, there were no changes in any parameters before and after the application (*p* > 0.05, Figures 1 and 2).

**FIGURE 3:**
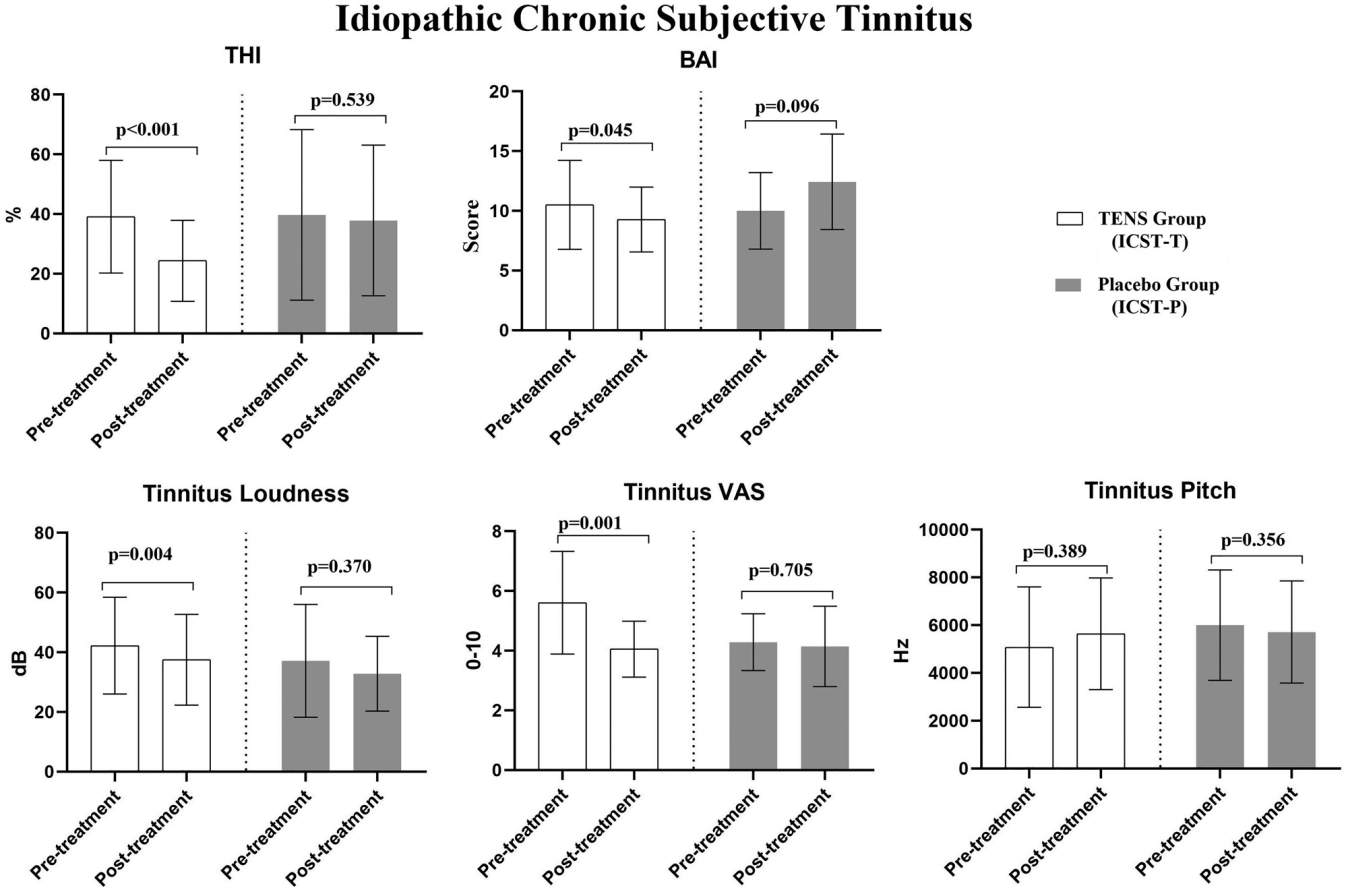
The effects of TENS (ICST‐T) and placebo (ICST‐P) on tinnitus handicap level, anxiety level, tinnitus‐VAS, neck pain‐VAS, and psychoacoustic characteristics in the idiopathic chronic subjective somatic tinnitus group.

**FIGURE 4:**
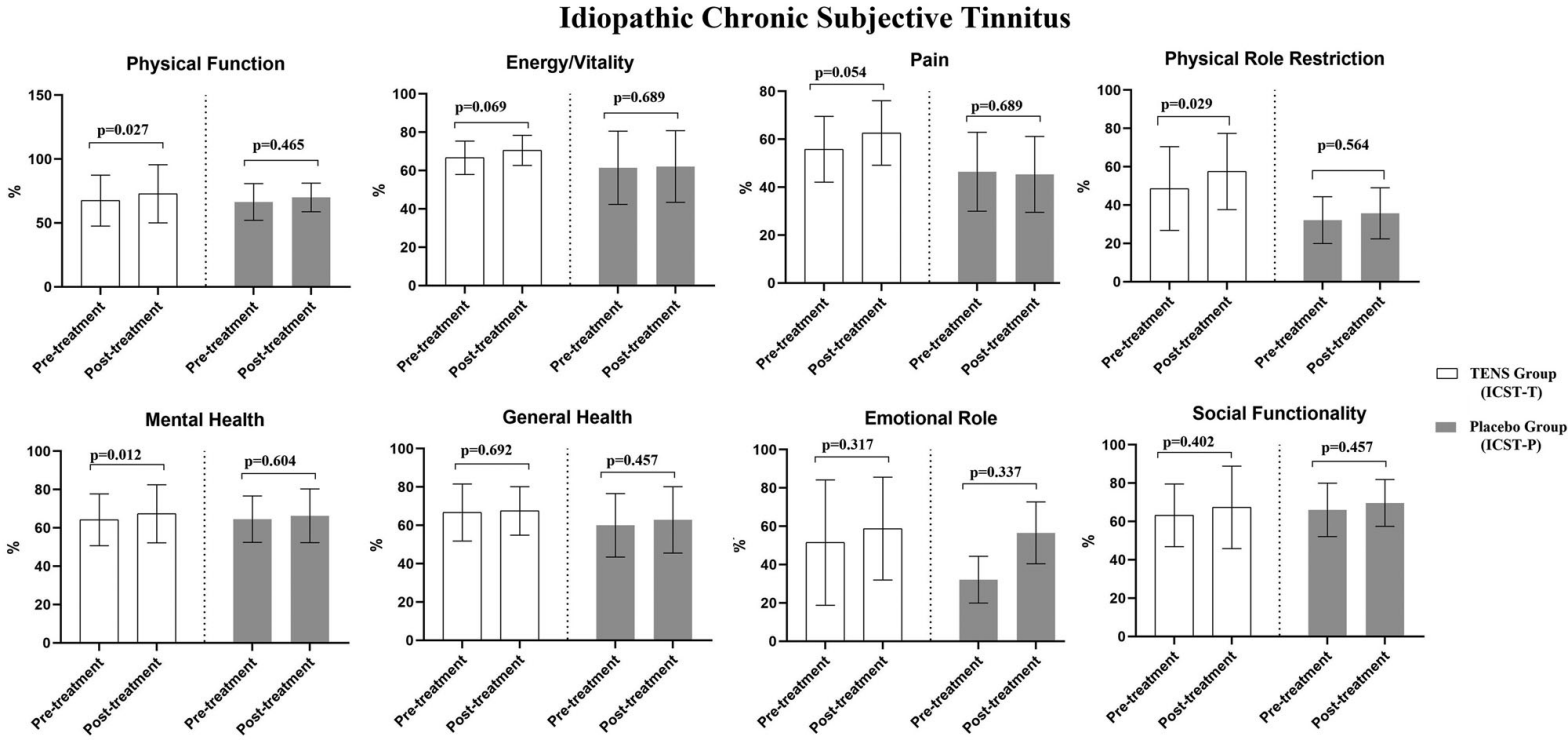
The effects of TENS (CST‐T) and placebo (CST‐P) on SF‐36 in idiopathic chronic subjective somatic tinnitus group.

In the ICST‐T group, TENS therapy resulted in significant improvements in THI, tinnitus loudness, BAI, VAS‐Tinnitus, as well as SF‐36/Physical Functioning, SF‐36/Role Physical, and SF‐36/Mental Health scores (*p* < 0.05). However, no changes were observed in other parameters before and after therapy (*p* > 0.05, Figures 3 and 4). In the ICST‐P group, no changes were found in any parameters before and after the application (*p *> 0.05, Figures 3 and 4).

## 4 Discussion

TENS has been used to treat pain, edema, ligament dysfunctions, and spinal diseases since the 19th century (Aydoğan et al. 2022). TENS is assumed to regulate tinnitus symptoms by increasing cochlear blood flow or regulating abnormal sensory signals based on the gate control theory (Luo et al. 2012; Steenerson and Cronin 2003). Tinnitus characteristics, etiology, pathophysiology, and psycho‐perceptual factors it creates in individuals are pretty diverse. Therefore, TENS therapy is unlikely to provide relief to all tinnitus patients (Hoare et al. 2016). C2 TENS therapy, which is recommended for tinnitus treatment, can also be used to treat chronic neck pain (Aydoğan et al. 2022; Deklerck et al. 2020). Therefore, this study assumed TENS may be more effective in individuals with neck‐related CST. We divided patients with CST and ICST into two groups and applied active TENS therapy to one group and placebo to the other group. In the CST group (CST‐T) with active treatment, THI, VAS‐tinnitus, VAS neck, and energy and pain subdimensions in the SF‐36 index improved significantly after treatment. In the ICST group (ICST‐T) with active treatment, THI, tinnitus loudness, BAI, VAS‐tinnitus, physical function, mental health, and physical role limitation subdimensions in the SF‐36 index improved significantly after treatment.

Neck pain, one of the most common musculoskeletal disorders, is a significant health problem for affected individuals. It significantly affects individuals socially, economically, and psychologically. Alghamdi et al. (2023) stated that there is a relationship between neck pain, and anxiety and depression and that as the severity of neck pain increases, depression and anxiety levels also increase. Similarly, another study revealed a relationship between musculoskeletal symptoms, especially neck problems, and psychological distress (Cho et al. 2003). In line with the literature, in our study, individuals with CST associated with neck pain had worse BAI, energy, and mental and general health scores than individuals with ICST.

Moreover, after TENS therapy, improvements were observed in tinnitus and neck pain scores of individuals in the CST‐T group, but no decrease in anxiety levels was observed. In the ICST‐T group, improvements were observed in both tinnitus and anxiety levels. Various hypotheses can explain this situation. The first of these is that anxiety related to neck pain may cause more severe and persistent psychological distress than anxiety related to tinnitus. Therefore, individuals with CTS related to neck pain may undergo extended TENS therapy or receive additional medical or behavioral treatments alongside TENS therapy. The other is that the TENS protocol applied to individuals may affect oxytocin, endorphin, and cortisol modulation. A study reported that high‐frequency TENS therapy is more effective (80–100 Hz and 350 µs) for anxiety related to pain (Sulu et al. 2022). In our study, we applied the TENS protocol (50 Hz and 50–100 µs) for tinnitus. Therefore, although this application reduced pain, it may not have affected anxiety.

Tinnitus can significantly diminish patients' quality of life. Salazar et al. (2019) found that 33% of tinnitus patients experience depression, highlighting the crucial need to evaluate the quality of life in these individuals. VAS, which is used as a valuable tool for rating pain, is also frequently used to assess tinnitus severity (De Meulemeester et al. 2023). Tinnitus can be graded between 0 and 10 with VAS. Thus, treatment outcomes can be easily evaluated with pretreatment and posttreatment applications. THI is a specialized tool designed to gauge the impact of tinnitus on daily living (Koçyiğit et al. 1999). It offers valuable insights by assessing the effect of tinnitus on life from the patient's perspective. Therefore, our study evaluated the effectiveness of TENS therapy with THI, VAS, and SF‐36 quality of life. A meta‐analysis study revealed that TENS treatment in individuals with tinnitus improved THI, VAS, and quality of life scores (Byun et al. 2020). The study showed that the overall mean reductions in THI and VAS were 7.55 out of 100 and 0.65 out of 10, respectively.

Although the effectiveness of TENS in tinnitus treatment has been indicated, there is limited information on which tinnitus etiology is more effective. Aydoğan et al. (2022) applied 20 sessions of C2 TENS therapy to individuals with chronic subjective tinnitus who had normal hearing and reported that TENS reduced the intensity of the tinnitus and improved the quality of life. Aydemir et al. (2006) applied TENS therapy to individuals with tinnitus of various etiologies (acoustic trauma, Meniere's disease, and idiopathic). The authors reported that TENS therapy can be used for tinnitus treatment and improves the quality of life in the individuals who receive it. However, they did not compare its effectiveness according to etiology. Another study investigated the effect of TENS on somatic tinnitus (Vanneste et al. 2010). The authors reported that 17.9% of the patients responded to TENS therapy and that there was an average improvement of 42.92% in these patients. In addition, the study reported that somatic tinnitus may have many causes and that treatment effectiveness may vary depending on the source of somatic tinnitus. Steenerson and Cronin (1999) applied probe electrical stimulation to tinnitus patients with various etiologies (sensorineural hearing loss, Meniere's disease, infection, head trauma, acoustic trauma, ototoxicity, and chemotherapy). The authors stated that electrical stimulation benefited approximately 50% of the patients. It was also noted that the patients with chemotherapy‐related tinnitus benefited the most, followed by infection and Meniere's disease. The study by Steenerson and Cronin (1999) was the only study we found in the literature that investigated the effectiveness of electrical stimulation according to tinnitus etiologies. Unlike Steenerson and Cronin (1999), our study included patients with somatic and idiopathic tinnitus who had normal hearing. The CST and ICST groups benefited from active TENS treatment compared to the placebo application. However, TENS therapy showed slightly more positive effects in the ICST‐T group compared to the CST‐T group in terms of anxiety, tinnitus severity, and SF‐36 (two vs. three subscales).

On the other hand, previous studies in the literature (Aydoğan et al. 2022; Vanneste et al. 2010; Aydemir et al. 2006) and the present study have primarily focused on the short‐term effects of TENS in tinnitus therapy, while its long‐term effects remain unknown. As is well established, TENS does not treat the underlying condition but rather alleviates acute and chronic pain symptomatically by activating the pain gate mechanism and/or the opioid system (Maeda et al. 2017). Moreover, patients with chronic pain have reported a decline in TENS efficacy with prolonged use (Jastreboff 2007). Thus, it remains unclear whether long‐term (> 3 months) TENS application influences tinnitus or whether the reduction in tinnitus perception following TENS treatment is sustained over time. Future studies investigating the long‐term effects of TENS on tinnitus could contribute to a better understanding of the underlying mechanisms of TENS‐tinnitus interaction.

There are two primary objectives in tinnitus therapies: to eliminate tinnitus or to help individuals habituate to the reactions triggered by tinnitus and subsequently adapt to its perception (Long 1991). Therefore, even if tinnitus cannot be entirely cured, improving patients' quality of life following therapy remains a crucial goal. The SF‐36 consists of eight subscales, providing a comprehensive assessment of quality of life. In our study, the significant improvements observed in the SF‐36 subscales of both the CST and ICST groups indicate the effectiveness of TENS therapy. This suggests that TENS therapy may be beneficial in reducing tinnitus perception and enhancing quality of life in patients with both CST and ICST. However, the primary limitation of our study is the small sample size in the placebo groups. Therefore, our study should be considered a pilot study investigating the effectiveness of TENS in CST and ICST.

Our placebo‐controlled comparative study confirms the assumption that tinnitus (somatic and non‐somatic) may be related to the DCN (Wang et al. 2009). The upper C2 nerves project to the spinal trigeminal nuclei, and electrical stimulation of C2 produces large potentials in the DCN. Stimulation of C2 produces an inhibition pattern in the principal cells of the DCN (Kanold and Young 2001). Therefore, TENS therapy can reduce tinnitus intensity and improve the quality of life in individuals with CST and ICST who have normal hearing. In future studies, the effectiveness of TENS can be compared with cases of tinnitus caused by hearing loss. In this way, it will be possible to understand which etiologies of tinnitus TENS may be effective, and tinnitus therapy strategies can be developed.

This study has several limitations. The first is the small sample size and the lack of homogeneity in the age distribution. Specifically, the discontinuation of therapy by individuals in the placebo group led to a reduction in the sample size. Future studies could investigate the short‐ and long‐term effects of TENS using larger and more homogeneous sample groups.

## 5 Conclusion

Although C2 TENS therapy is considered a more effective treatment method in patients with neck‐related CST, our placebo‐controlled comparative study shows that C2 TENS therapy can be used effectively to suppress tinnitus and improve quality of life in both CST and ICST patients. Future studies may contribute to developing treatment strategies by examining the long‐term effects of this treatment method and its effectiveness in different tinnitus subgroups in more detail.

## Author Contributions

**Emre Soylemez**: methodology, resources, visualization, writing – original draft, funding acquisition, writing – review and editing. **Aydin Sinan Apaydin**: conceptualization, validation, data curation, writing – original draft, writing – review and editing. **Zehra Aydoğan**: resources, supervision, data curation, project administration. **Neslihan Hazal Şen**: conceptualization, investigation, data curation, formal analysis, validation. **Murat Yasar**: methodology, validation, visualization, software, resources, supervision. **Ömer Gökay Argadal**: formal analysis, project administration, resources, supervision.

## Ethics Statement

We obtained both verbal and written consent from all participants in accordance with the Declaration of Helsinki. Ethical approval was obtained for the study from the Kastamonu University Clinical Research and Evaluation Commission (2024‐KAEK‐39).

## Consent

Written and verbal consent was obtained from the participants.

## Conflicts of Interest

The authors declare no conflicts of interest.

### Peer Review

The peer review history for this article is available at https://publons.com/publon/10.1002/brb3.70429

## Data Availability

The data that support the findings of this study are available on request from the corresponding author. The data are not publicly available due to privacy or ethical restrictions. The data collected and presented in the current study are not available publicly or by request due to privacy and ethical concerns.
